# Independent control of organ number and distribution pattern in rice panicle

**DOI:** 10.3389/fpls.2023.1119770

**Published:** 2023-02-07

**Authors:** Eiji Yamamoto, Shiori Yabe, Mayuko Inari-Ikeda, Hideki Yoshida, Yoichi Morinaka, Makoto Matsuoka, Hidemi Kitano

**Affiliations:** ^1^ Graduate School of Agriculture, Meiji University, Kawasaki, Kanagawa, Japan; ^2^ Institute of Crop Science, National Agriculture and Food Research Organization, Tsukuba, Ibaraki, Japan; ^3^ Bioscience and Biotechnology Center, Nagoya University, Nagoya, Aichi, Japan; ^4^ Institute of Fermentation Sciences, Fukushima University, Fukushima, Fukushima, Japan; ^5^ Department of Sustainable Agri-Culture, Fukui Prefectural University, Awara, Fukui, Japan

**Keywords:** rice, panicle, QTL, organ distribution pattern, morphometrics

## Abstract

As the determinants of yield products, rice panicle traits are important targets for breeding. Despite their importance in grain filling and subsequent yield productivity, knowledge on the organ distribution pattern in rice panicles is limited owing to the lack of objective evaluation methods. In this study, we developed a method for quantifying rice panicle organ distribution patterns. To validate our method for practical application in biology, we integrated this method into a quantitative trait locus (QTL) analysis and identified QTLs for panicle organ distribution patterns in rice. Interestingly, *Grain number 1* (*Gn1*), a major QTL of organ number, was not identified as a QTL for distribution pattern, indicating that the number and distribution of panicle organs are independently controlled. This study provides insight into rice panicle organ distribution patterns that will help improve breeding targeting rice panicle architecture.

## 1 Introduction

**Figure 1:**
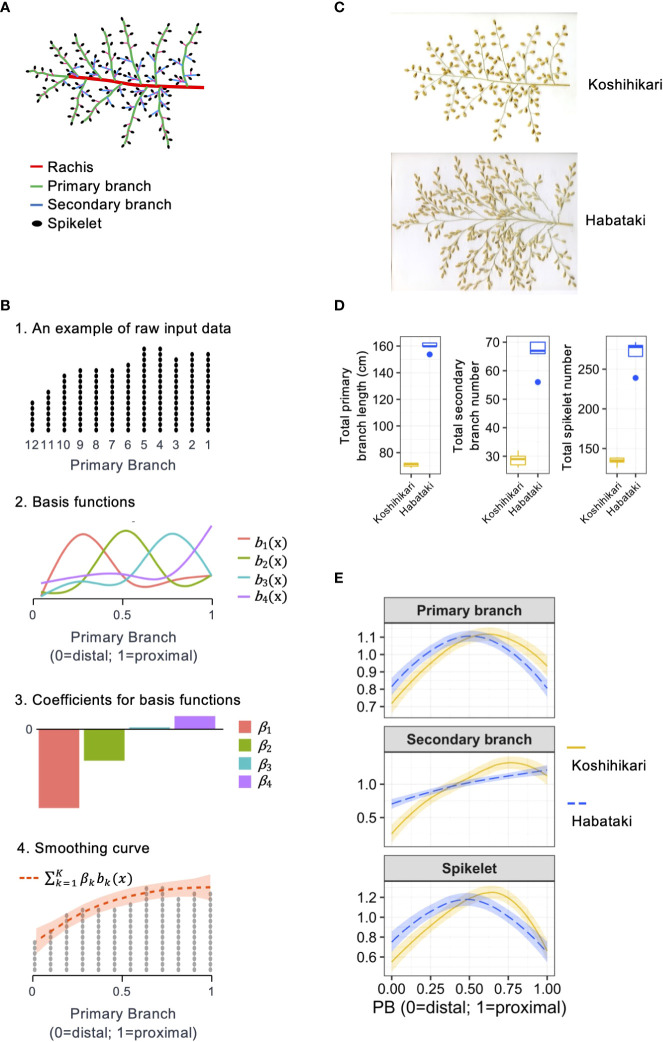
Method for the quantitative description of organ distribution patterns in rice panicle. **(A)** Schematic representation of the rice panicle architecture. **(B)** Schematic representation of the method used to describe the organ distribution pattern using a cubic smoothing spline. **(C)** Panicle morphologies of Koshihikari and Habataki. **(D)** Boxplots for phenotypic values of Koshihikari and Habataki. Box edges represent 0.25 and 0.75 quantiles with median values indicated by bold lines. Whiskers extend to data no more than 1.5 times the interquartile range, and remaining data are indicated by dots. **(E)** Smoothing curves of organ distribution patterns drawn for Koshihikari (yellow solid lines) and Habataki (blue dashed lines). The shaded areas represent the 95% confidence bands.

Panicle is the inflorescence of rice and has a complicated architecture consisting of several types of organs (Ikeda et al., 2004) (**Figure 1A**). The primary and higher-order branches can directly generate spikelets, which are the flowers of rice. When a spikelet is pollinated and receives enough source for filling, it becomes a grain, which is the yield product.

Rice panicle architecture is an important target in genetics, molecular biology, and breeding studies (Xing and Zhang, 2010; Li et al., 2021). For example, a natural allele of *Grain number 1* (*Gn1*) increases the number of spikelets (Ashikari et al., 2005). *ABERRANT PANICLE ORGANIZATION 1* (*APO1*) was first identified as a gene involved in inflorescence meristem activity and longevity *via* mutant analyses (Ikeda-Kawakatsu et al., 2009). Later, the natural allele *Harvest Index 1* (*HI1*)/*STRONG CULM2* (*SCM2*) was identified as a quantitative trait locus (QTL) for the number of spikelets (Ookawa et al., 2010; Terao et al., 2010).

In addition to the number of organs, grain-filling efficiency is another essential factor for rice yield because poor grain filling results in low yield, even if the panicle has a large number of spikelets (Yang and Zhang, 2010; Ohsumi et al., 2011; Parida et al., 2022). Breeders and geneticists have found variations in the organ distribution patterns of the rice panicle (Matsuba, 1991; Yamaki et al., 2010), and many studies have reported the relationship between grain-filling efficiency and distribution pattern of organs (Seki et al., 2011; Yoshinaga et al., 2013). Thus, the number of organs and their distribution patterns affect the sink capacity and source allocation, respectively. Therefore, the design of organ distribution patterns is an important breeding target for achieving high grain-filling efficiency and yield (Xu et al., 2005; Peng et al., 2008). However, the organ distribution pattern has rarely been genetically analyzed because of the absence of a quantitative description method.

Similar to the organ distribution pattern in rice panicle, there are plant phenotypes that are difficult to describe in quantitative manner. One representative is the shape of organs (e.g., leaves and roots) (Bucksch et al., 2017). Morphometric descriptors, such as elliptic Fourier analysis, can be used for quantitative description (Iwata and Ukai, 2002). In this approach, the contour of the shape was mathematically described using Fourier series expansions. Subsequently, the coefficients of expansions were used as the descriptors. Another example is the density distribution or histogram of measurements from a sample (e.g., grain weight distribution). A mixture of probability densities is useful for describing such phenotypes (Yabe et al., 2018). Due to the difficulty in understanding the original shape using raw descriptors, summary variables or principal component scores are used to recognize patterns that the descriptors represent (Yoshioka et al., 2005). The application of such approaches has contributed to various biological studies, including identification of genetic factors for organ development (Chitwood et al., 2014), molecular functional analysis of developmental pattern dynamism (Chitwood et al., 2012), and genomic prediction of organ shape (Iwata et al., 2010). However, there have been no reports of such morphometric approaches enabling the identification of agronomically important QTLs.

In this study, we developed a method for quantifying rice panicle organ distribution patterns. To validate our method for practical application in biology, we integrated this method into a quantitative trait locus (QTL) analysis and identified QTLs for panicle organ distribution patterns in rice.

## 2 Materials and methods

### 2.1 Plant materials

We used Koshihikari (*Oryza sativa* ssp. *japonica*) and Habataki (*O. sativa* ssp. *indica*) as genetic resources in this study. A total of 75 backcrossed inbred lines (BILs) were developed *via* self-pollination after crossing Koshihikari/Habtaki F_1_ and Koshihikari. Near-isogenic lines (NILs) with introgression of the Habataki genome in the Koshihikari genetic background were developed *via* three to five rounds of backcrossing and marker-assisted selection (Ashikari et al., 2005; Ookawa et al., 2010).

### 2.2 Genotyping

Total DNA was extracted from leaf samples using the DNeasy Plant Mini Kit (QIAGEN, Hilden, Germany). Genotyping was performed using the Illumina GoldenGate BeadArray technology platform (Illumina, Inc., San Diego, CA, USA) with single-nucleotide polymorphism markers provided by Nagasaki et al. (2010). The number of polymorphic markers were 878. The genotype data are given in supplementary material.

### 2.3 Phenotyping

The plant materials were grown in a paddy field at the Togo Field for Science and Education at Nagoya University in 2015. Seeds were immersed in water for 2 days and then sown in a nursery bed. One-month-old seedlings were transplanted to a paddy field with a spacing of 20 × 35 cm. The panicle of the main culm of each plant was used for phenotyping. To evaluate the organ distribution patterns, the primary branch length, secondary branch number, and spikelet number on each primary branch were measured manually. The data are the average of three and five plants for BILs and others, respectively. The phenotype data are given in supplementary material.

### 2.4 Description of the organ distribution pattern

To describe the organ distribution pattern in the panicle, the measured values were arranged along the order of the primary branches on the rachis (**Figures 1A, B**). To evaluate the organ distribution pattern regardless of the primary branch number, the order of the primary branches was adjusted from 0 (for primary branch at distal end) to 1 (for primary branch at proximal end) (**Figure 1B**). According to this adjusted primary branch position, the distributions of the primary branch length, secondary branch number, and spikelet number were interpolated using smooth modeling. For smooth modeling, we used a cubic smoothing spline:


(1) $$y_{i}=\alpha +\sum_{k=1}^{K}\beta_{k}b_{k}\left(x_{i}\right)+\epsilon_{i}$$

where *y_i_* is a response variable (each primary branch length, secondary branch number, and spikelet number), *x_i_* is a covariate (adjusted position of the primary branch) at the *i*th data point, *b_k_* () is the *k*th basis of the cubic spline (**Figure 1B**), *α* and *β_k_* are coefficients (**Figure 1B**), and *ε* is a random error. The model was fitted separately for each trait (i.e., primary branch length, secondary branch number, and spikelet number). The degrees of freedom (df) of the smoothing spline function were selected from 3–5 by using the sum of global cross validation (GCV) values over all BILs in each trait. The maximum value of 5 was determined because this value was the minimum number of primary branches in the panicles analyzed in this study. The position of the knots was fixed among the panicles in the estimation trials with the same df to enable direct comparison of the coefficients from the same set of basis functions between the panicles. As a result of GCV, df was determined as 4, 5, and 5 for primary branch length, secondary branch number, and spikelet number, respectively. The calculations were performed using the function “gam” in the *R package mgcv* (Wood, 2011). To use the coefficients (*α* and *β*) as descriptors of the organ distribution pattern regardless of the size, we standardized the cumulative distribution function from 0 to 1 as 1. These adjusted coefficients were used as descriptors of the organ distribution pattern (i.e. phenotypic value). To provide an intuitive understanding of the organ distribution patterns, we also performed PCA using these adjusted coefficients as variables and used the principal component scores as feature quantities. PCA and derivation of the principal component scores were performed using the R basic function “prcomp” (https://www.r-project.org/). An R script used for these analyses is given in supplementary material.

### 2.5 Heritability

The narrow-sense heritability ( $\hat{h^{2}}$ ) was estimated using equation 2:


(2) $${\hat{h}}^{2}={\hat{\sigma }}_{G}^{2}/\left({\hat{\sigma }}_{G}^{2}+{\hat{\sigma }}_{\epsilon }^{2}\right)$$

where ${\hat{\sigma }}_{G}^{2}$ and ${\hat{\sigma }}_{\epsilon }^{2}$ are the genetic and error variances, respectively. These variance components were estimated by solving equation 3:


(3) $$V=G{\hat{\sigma }}_{G}^{2}+I{\hat{\sigma }}_{\epsilon }^{2}$$

where *V* is the phenotypic variance; *I* is an identity matrix; *G* is the genetic relationship matrix calculated by function “A.mat” in the *R package rrBLUP* version 4.3 (Endelman and Jannink, 2012). The solution of equation (3) was obtained by using function “mixed.solve” in the *R package rrBLUP* version 4.3 (Endelman, 2011).

### 2.6 QTL mapping

QTL mapping was performed using functions in the *R package qtl* version 1.42-8 (Broman et al., 2003). The linkage map positions of the markers were estimated based on kosambi map function using the function “read.cross” with the arguments map.function=“kosambi”, BC.gen=1 and F.gen=7. QTL mapping was performed using function “scanone” with the arguments model=“normal” and method=“em.” The logarithm of the odds score significance threshold was determined using 1,000 permutations.

## 3 Results

### 3.1 Description of the organ distribution pattern

For quantitative description, we described the organ distribution pattern in a rice panicle as a non-linear function using the order of primary branches along the rachis and the number or length of organs on each primary branch as the explanatory and response variables, respectively (**Figure 1B**). This method describes the organ distribution pattern as if it is smoothing the histogram of the number or length of organs on the relative position of primary branches (**Figure 1B**). For this method, cubic smoothing splines (Wood, 2011) were used. We used common basis functions to enable a direct comparison of the parameters from the panicles with different primary branch numbers. In addition, the smoothing curves were standardized to make the integral equal to 1 to eliminate the effect of size factors, such as the total number or length of organs. Using this method, we described the organ distribution patterns of two rice varieties, Koshihikari and Habataki (**Figures 1C–E**). Regarding the distribution patterns of primary branch length and spikelet number, Koshihikari and Habataki showed proximally and distally laid patterns, respectively (**Figure 1E**). In the distribution pattern of secondary branch number, Koshihikari and Habataki showed curvilinear and linear distribution patterns, respectively (**Figure 1E**).

**Figure 2:**
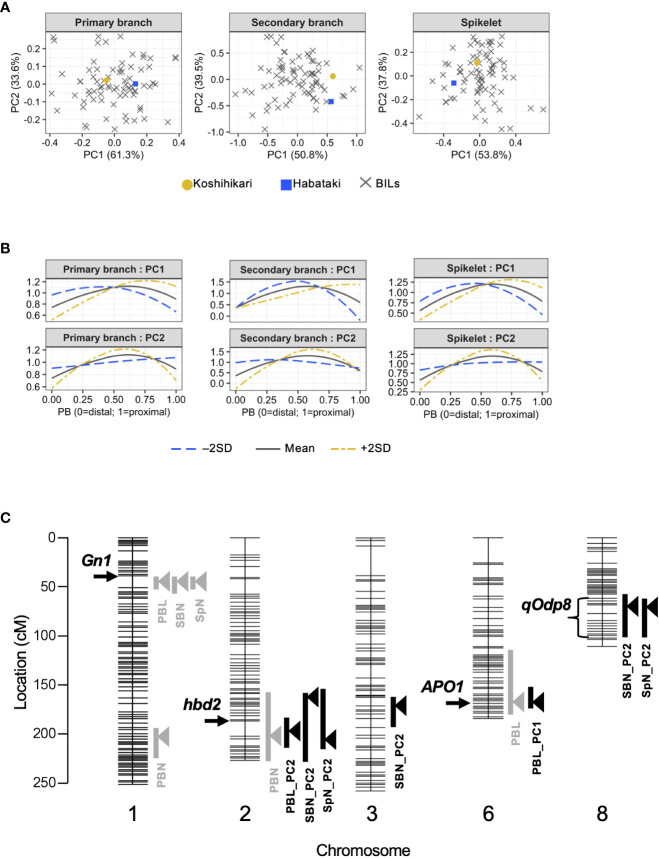
Quantitative trait locus (QTL) analysis of organ distribution patterns. **(A)** Organ distribution patterns in backcrossed inbred lines (BILs) derived from Koshihikari and Habataki. **(B)** Effect of each principal component (PC). Estimated organ distribution patterns were recalculated for mean ± 2 standard deviations (SD) of the PC. **(C)** Chromosomal location of the QTLs detected in this study. Horizontal lines indicate the positions of DNA markers. The arrowheads and bars on the right side of each chromosome indicate the position of the highest peak and –2 logarithm of the odds (LOD) confidential interval, respectively. The arrows and bracket on the left side of each chromosome indicate the position of previously identified genes and a novel QTL, respectively. PBN, primary branch number; PBL, total primary branch length; SBN, total secondary branch number; SpN, total spikelet number; PBL_PCx, PCx of distribution pattern of primary branch length; SBN_PCx, PCx of distribution pattern of secondary branch number; SpN_PCx, PCx of distribution pattern of spikelet number.

To investigate whether organ distribution patterns are under genetic control, we used BILs derived from a cross between Koshihikari/Habataki F_1_ and Koshihikari (**Figure 2A**). We performed principal component analysis (PCA) to summarize the information contained in the spline coefficients. In all traits of the distribution pattern (i.e., PCs of primary branch length, secondary branch number and spikelet number), the proportion of variance explained by PC1 and PC2 was explicitly high, with the cumulative contribution of these two PCs reaching 90% (**Figure 2A**, **Table 1**). Therefore, we focused on PC1 and PC2 for further analyses. To recognize the effect of each PC, we recalculated the spline coefficients, letting the scores on a particular PC equal to the mean ± standard deviation, while fixing the other PC scores as the means, and reconstructed the smoothing curve (**Figure 2B**). PC1 and PC2 showed similar features for all traits. PC1 represented proximally or distally laid patterns similar to the differences in primary branch length and spikelet number between Koshihikari and Habataki (**Figure 1E**). PC2 represented a curvilinear or linear pattern that resembled the difference in the number of secondary branches between Koshihikari and Habataki (**Figures 1E**, **2B**). Indeed, the difference in PC2 between Koshihikari and Habataki was the largest in secondary branch number (**Figure 2A**). Then we estimated narrow-sense heritability of the traits analyzed in this study (**Table 2**). The result suggested that organ distribution patterns are under genetic control.

**Table 1:** Proportion of variance explained by each principal component (PC).

Trait	df of smoothing splines	Proportion of variance explained (%)				
		PC1	PC2	PC3	PC4	PC5
Primary branch length	4	61.3	33.6	5.1	0.0	–
Secondary branch number	5	50.8	39.5	6.6	3.1	0.0
Spikelet number	5	53.8	37.8	7.0	1.4	0.0

**Table 2:** Estimated narrow-sense heritability.

Trait	$\hat{h^{2}}$
Primary branch number	0.364
Total primary branch length	0.911
Total secondary branch number	0.618
Total spikelet number	0.769
Primary branch length PC1	0.350
Primary branch length PC2	0.347
Secondary branch number PC1	0.027
Secondary branch number PC2	0.340
Spikelet number PC1	0.154
Spikelet number PC2	0.348

### 3.2 QTL mapping

In QTL analysis of the number or length of organs, *Gn1* was detected as a major QTL for total primary branch length, total secondary branch number, and total spikelet number (Ashikari et al., 2005) (**Figure 2C**, **Table 3**). However, unlike previous studies (Ookawa et al., 2010; Terao et al., 2010), *APO1* was not detected as a QTL for total spikelet number (**Figure 2C**). Alternatively, *APO1* was detected as a QTL for the total primary branch length (**Figure 2C**, **Table 3**). In addition to the QTLs identified in previous studies, we detected a novel QTL for the primary branch number on chromosome 1 (**Figure 2C**, **Table 3**). In the QTL analysis for organ distribution patterns, we detected a QTL for PC1 of primary branch length in a region that included *APO1* (**Figure 2C**, **Table 3**). We refer to this QTL as *APO1* hereinafter because the effects have been confirmed in NIL_*APO1* (see the next section). The QTL on chromosome 8 was associated with PC2 of secondary branch and spikelet number (**Figure 2C**, **Table 3**). We named this QTL *qOdp8* (QTL for the organ distribution pattern on chromosome 8). As for the PC2 of secondary branch number, another QTL was detected on chromosome 3 (**Figure 2C**, **Table 3**). Interestingly, *Gn1* was not associated with organ distribution patterns despite its major effects on the number or length of organs (**Figure 2C**, **Table 3**). Additionally, we detected a QTL involved in multiple traits on chromosome 2 (**Figure 2C**, **Table 3**). This QTL region included *hybrid breakdown 2* (*hbd2*), a gene involved in hybrid weakness (Yamamoto et al., 2007). Among the detected QTLs, we focused on three QTLs: *Gn1*, *APO1*, and *qOdp8* (**Figure 2C**). *Gn1* and *APO1* were selected because these QTLs have been well analyzed in previous studies (Ashikari et al., 2005; Ookawa et al., 2010; Terao et al., 2010). In addition, *qOdp8* was selected because the QTL was associated with two organ distribution patterns, namely the secondary branch and spikelet number, which convinced us that the QTL was not a false-positive. However, as we could not discern whether the effects of this QTL were side effects of hybrid weakness, we removed *hbd2* from further analyses.

**Table 3:** Estimated effects of quantitative trait loci (QTLs).

Trait	Chr	Nearest marker	Position (cM)	LOD	PVE	Koshihikari-allele	Habataki-allele
Primary branch number	1	Geno_NIAS04066	202.3	3.960	0.216	13.033 ± 0.269	10.728 ± 0.431
Primary branch number	2	Geno_NIAS10171	204.9	3.054	0.171	12.867 ± 0.264	10.618 ± 0.507
Total primary branch length	6	Geno_NIAS26448	168.7	0.261	0.016	12.241 ± 0.288	12.927 ± 0.553
Total primary branch length	1	Geno_NIAS01002	45.0	8.737	0.387	92.583 ± 2.253	121.571 ± 4.343
Total secondary branch number	1	Geno_NIAS01002	45.0	6.904	0.317	30.082 ± 1.520	46.694 ± 2.778
Total spikelet number	1	Geno_NIAS01002	45.0	8.558	0.365	161.982 ± 6.360	239.123 ± 11.797
Primary branch length PC1	6	Geno_NIAS09083	167.5	3.738	0.187	0.017 ± 0.025	–0.024 ± 0.034
Primary branch length PC2	2	Geno_NIAS09998	197.5	4.608	0.230	0.016 ± 0.017	–0.042 ± 0.028
Secondary branch number PC2	2	Geno_NIAS09051	162.5	4.519	0.224	0.038 ± 0.052	–0.053 ± 0.068
Secondary branch number PC2	3	Geno_NIAS14274	171.2	3.042	0.170	–0.083 ± 0.044	0.259 ± 0.075
Secondary branch number PC2	8	Geno_NIAS32135	68.4	3.026	0.170	0.075 ± 0.042	–0.306 ± 0.088
Spikelet number PC2	2	Geno_NIAS10171	207.5	3.927	0.200	0.035 ± 0.025	–0.098 ± 0.042
Spikelet number PC2	8	Geno_NIAS32135	68.4	3.055	0.171	0.040 ± 0.023	–0.167 ± 0.047

LOD, Logarithm of odds score.

PVE, Proportion of variance explained.

### 3.3 Confirmation of QTL effects

**Figure 3:**
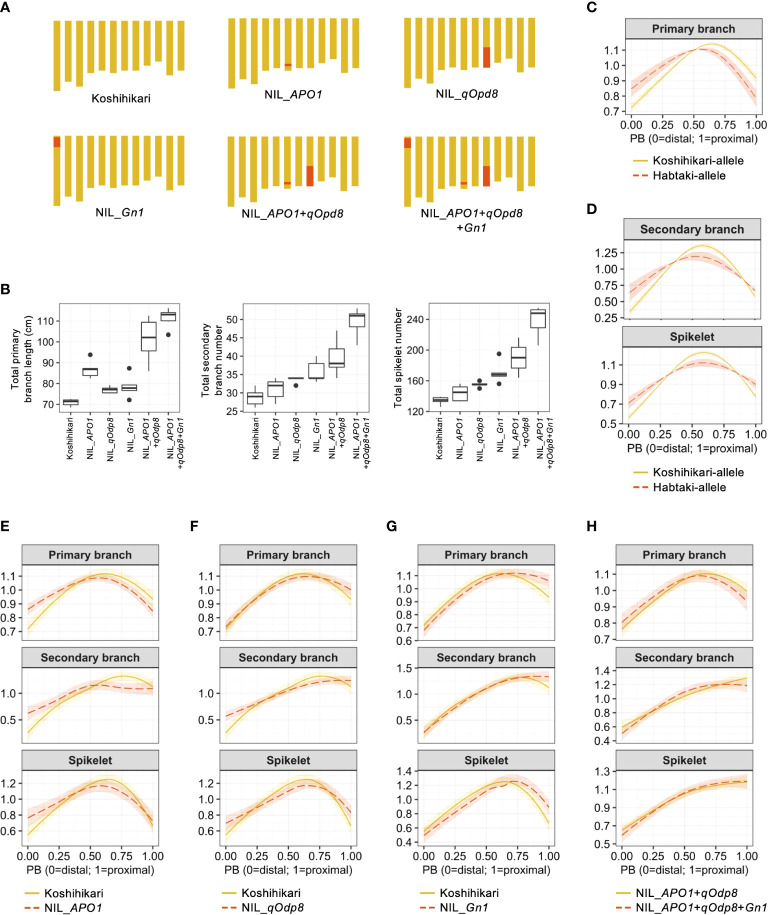
Confirmation of QTL effects using near-isogenic lines (NILs). **(A)** Graphical genotypes of NILs used in this study. Yellow and red areas indicate the genomic regions derived from Koshihikari and Habataki, respectively. **(B)** Boxplots of the phenotypic values of NILs. Box edges represent 0.25 and 0.75 quantiles with median values shown by bold lines. Whiskers extend to data no more than 1.5 times the interquartile range, and remaining data are indicated by dots. **(C)** Estimated effect of *APO1* on the organ distribution pattern. **(D)** Estimated effects of *qOdp8* on the organ distribution patterns. **(E)** Smoothing curves of organ distribution patterns for Koshihikari (yellow solid lines) and NIL_*APO1* (red dashed lines). **(F)** Smoothing curves of organ distribution patterns for Koshihikari (yellow solid lines) and NIL_*qOpd8* (red dashed lines). **(G)** Smoothing curves of organ distribution patterns for Koshihikari (yellow solid lines) and NIL_*Gn1* (red dashed lines). **(H)** Smoothing curves of organ distribution patterns for NIL_*APO1*+*qOpd8* (yellow solid lines) and NIL_*APO1*+*qOpd8*+*Gn1* (red dashed lines). The shaded areas in **C–H** represent the 95% confidence bands.

To compare the estimated QTL effects and NIL phenotypes (**Figures 3A, B**), we recalculated the organ distribution patterns for each QTL by assigning the estimated allelic effects to the cubic smoothing spline functions (**Figures 3C, D**; **Table 3**). The effect of *APO1* on total primary branch length was confirmed in NIL_*APO1* that was developed in a previous study (Ookawa et al., 2010) (**Figures 3A, B**). Unlike previous studies (Ookawa et al., 2010; Terao et al., 2010), *APO1* was not detected in the total secondary branch and spikelet number in our QTL analysis (**Figure 2C**). However, an increase in secondary branches and spikelets was observed in NIL (**Figure 3B**). Reconstruction of the estimated *APO1* effect on organ distribution pattern suggested that the Habataki-*APO1* allele caused a distally laid primary branch length pattern (**Figure 3C**). NIL_*APO1* showed higher values in the distal position of the primary branch length pattern than Koshihikari (**Figure 3E**). Thus, NIL_*APO1* reproduces the estimated *APO1* effect. In addition, NIL_*APO1* affected the distribution pattern of secondary branches and spikelets, but these effects were not significant in QTL analysis (**Figures 2C**, **3E**). Although there were no significant associations between *qOdp8* and total primary branch length and secondary branch number (**Figure 2C**), NIL_*qOdp8* showed higher values for these traits (**Figure 3B**). The effects of reconstructed *qOdp8* on the organ distribution patterns suggested that the Habataki-*qOdp8* allele caused a distally laid secondary branch and spikelet pattern (**Figure 3D**). In the spikelet distribution pattern, the NIL_*qOdp8* phenotype was consistent with the estimated effect (**Figure 3F**). As for the distribution pattern of secondary branches, NIL_*qOdp8* showed a linear pattern, whereas Koshihikari showed a curvilinear pattern (**Figure 3F**). Because *qOdp8* was detected for the PC2 of secondary branch distribution pattern (**Figure 2C**) and the feature represented by PC2 was linear or curvilinear (**Figure 2B**), the observation of NIL_*qOdp8* confirmed that *qOdp8* is a QTL for PC2. Moreover, in the distribution pattern of the primary branch length, 95% confidence bands of the curves overlapped in the entire region between Koshihikari and NIL_*qOdp8* (**Figure 3F**). This result was consistent with the QTL analysis results that the effect of *qOdp8* on the primary branch length distribution pattern was insignificant. Thus, the estimated *qOdp8* effects on organ distribution patterns were confirmed in NIL_*qOdp8*. One of the most interesting findings of our QTL analysis was that *Gn1* had little effect on organ distribution patterns despite its large impact on the number and length of organs (**Figure 3B**). To confirm these results, two comparisons were made. One was a comparison between Koshihikari and NIL_*Gn1* that was developed in a previous study (Ashikari et al., 2005), and the other was between NIL_*APO1*+*qOdp8* and NIL_*APO1*+*qOdp8*+*Gn1* (**Figures 3A, B**). Because Koshihikari and NIL_*APO1*+*qOdp8* have different organ distribution patterns, the use of these lines aids in determining whether the estimated effect of *Gn1* is specific to an organ distribution pattern or the genetic background. In both comparisons, the addition of *Gn1* did not significantly affect the organ distribution pattern (i.e., 95% confidential bands overlapped in the entire region, **Figures 3G, H**). These results confirm that *Gn1* has little effect on the organ distribution pattern.

## 4 Discussion

In this study, we developed a method to quantitatively describe the organ distribution patterns in rice panicles (**Figure 1**). We performed QTL analysis to prove the validity of our method (**Figure 2**). The effects of QTLs that were involved in organ distribution patterns were confirmed *via* phenotypic observation of the NILs (**Figure 3**). While *APO1* and *qOdp8* affected the organ distribution patterns (**Figure 3**), *Gn1* showed little effect on organ distribution patterns despite its significant effects on the number and length of organs (**Figures 3B, G, H**). These results indicate that there are mechanisms that independently control the number of organs and distribution patterns. Although this study focused on the development of a method to describe organ distribution patterns and its application for QTL analysis, some molecular insights are also available from the natural allelic variants identified in the QTLs. *Gn1* consists of several linked QTLs (Ashikari et al., 2005). Among these QTLs, *Gn1a* encodes a cytokinin degradation enzyme. The low activity of the Habataki-*Gn1a* allele results in increased cytokinin content in the inflorescence meristem, which subsequently increases meristem activity (Ashikari et al., 2005). The causal gene of *APO1* is known to regulate inflorescence meristem activity and longevity (Ikeda-Kawakatsu et al., 2009). Because *Gn1* showed little effect, while *APO1* had a significant effect on the organ distribution patterns, the meristem longevity was suggested to be a key of organ distribution pattern in rice panicles, while meristem activity was not as important. Since organ distribution patterns are important for high grain-filling efficiency and yield, the method developed and the results of this study will be useful for future rice breeding (Xu et al., 2005; Peng et al., 2008; Parida et al., 2022). One drawback of our method is that it requires significant effort to measure the number and length of the organs. However, recent advances in high-throughput phenotyping technologies will make our approach feasible (Crowell et al., 2014).

## Data availability statement

The original contributions presented in the study are included in the article/**Supplementary Material**. Further inquiries can be directed to the corresponding author.

## Author contributions

EY, SY, MM, and HK designed the study. EY and SY analyzed the data. MI-I, HY, and YM performed the genotyping and phenotyping of materials. EY and SY wrote the paper. All authors reviewed and approved the final manuscript.

## Supplementary material

SUPPLEMENTARY DATA SHEET 1 The genotype data used in this study.

SUPPLEMENTARY DATA SHEET 2 The phenotype data used in this study.

SUPPLEMENTARY DATA SHEET 3 An R script used in this study.

## References

[B1] Ashikari M. Sakakibara H. Lin S. Yamamoto T. Takashi T. Nishimura A.. (2005). Cytokinin oxidase regulates rice grain production. Science 309, 741–745. doi: 10.1126/science.1113373 15976269

[B2] Broman K. W. Wu H. Sen S. Churchill G. A. (2003). R/qtl: QTL mapping in experimental crosses. Bioinformatics 19, 889–890. doi: 10.1093/bioinformatics/btg112 12724300

[B3] Bucksch A. Atta-Boateng A. Azihou A. F. Battogtokh D. Baumgartner A. Binder B. M.. (2017). Morphological plant modeling: Unleashing geometric and topological potential within the plant sciences. Front. Plant Sci. 8. doi: 10.3389/fpls.2017.00900 PMC5465304 28659934

[B4] Chitwood D. H. Headland L. R. Ranjan A. Martinez C. C. Braybrook S. A. Koenig D. P.. (2012). Leaf asymmetry as a developmental constraint imposed by auxin-dependent phyllotactic patterning. Plant Cell 24, 2318–2327. doi: 10.1105/tpc.112.098798 22722959 PMC3406905

[B5] Chitwood D. H. Ranjan A. Martinez C. C. Headland L. R. Thiem T. Kumar R.. (2014). A modern ampelography: A genetic basis for leaf shape and venation patterning in grape. Plant Physiol. 164, 259–272. doi: 10.1104/pp.113.229708 24285849 PMC3875807

[B6] Crowell S. Falcão A. X. Shah A. Wilson Z. Greenberg A. J. McCouch S. R. (2014). High-resolution inflorescence phenotyping using a novel image-analysis pipeline, PANorama. Plant Physiol. 165, 479–495. doi: 10.1104/pp.114.238626 24696519 PMC4044845

[B7] Endelman J. B. (2011). Ridge regression and other kernels for genomic selection with *R package* rrBLUP. Plant Genome 4, 250–255. doi: 10.3835/plantgenome2011.08.0024

[B8] Endelman J. B. Jannink J. L. (2012). Shrinkage estimation of the realized relationship matrix. G3 (Bethesda) 2, 1405–1413. doi: 10.1534/g3.112.004259 23173092 PMC3484671

[B9] Ikeda-Kawakatsu K. Yasuno N. Oikawa T. Iida S. Nagato Y. Maekawa M.. (2009). Expression level of *ABERRANT PANICLE ORGANIZATION1* determines rice inflorescence form through control of cell proliferation in the meristem. Plant Physiol. 150, 736–747. doi: 10.1104/pp.109.136739 19386809 PMC2689948

[B10] Ikeda K. Sunohara H. Nagato Y. (2004). Developmental course of inflorescence and spikelet in rice. Breed. Sci. 54, 147–156. doi: 10.1270/jsbbs.54.147

[B11] Iwata H. Ebana K. Uga Y. Hayashi T. Jannink J. L. (2010). Genome-wide association study of grain shape variation among *Oryza sativa* l. germplasms based on elliptic Fourier analysis. Mol. Breed. 25, 203–215. doi: 10.1007/s11032-009-9319-2

[B12] Iwata H. Ukai Y. (2002). SHAPE: A computer program package for quantitative evaluation of biological shapes based on elliptic Fourier descriptors. J. Hered. 93, 384–385. doi: 10.1093/jhered/93.5.384 12547931

[B13] Li G. Zhang H. Li J. Zhang Z. Li Z. (2021). Genetic control of panicle architecture in rice. Crop J. 9, 590–597. doi: 10.1016/j.cj.2021.02.004

[B14] Matsuba K. (1991). The morphogenetic mechanism of formation of the panicle branching system in rice plants (*Oryza sativa* l.). Bull. Chugoku Natl. Agric. Exp. Stn. 9, 11–58.

[B15] Nagasaki H. Ebana K. Shibaya T. Yonemaru J. Yano M. (2010). Core single-nucleotide polymorphisms–a tool for genetic analysis of the Japanese rice population. Breed Sci. 60, 648–655. doi: 10.1270/jsbbs.60.648

[B16] Ohsumi A. Takai T. Ida M. Yamamoto T. Arai-Sanoh Y. Yano M.. (2011). Evaluation of yield performance in rice near-isogenic lines with increased spikelet number. Field Crop Res. 120, 68–75. doi: 10.1016/j.fcr.2010.08.013

[B17] Ookawa T. Hobo T. Yano M. Murata K. Ando T. Miura H.. (2010). New approach for rice improvement using a pleiotropic QTL gene for lodging resistance and yield. Nat. Commun. 1, 132. doi: 10.1038/ncomms1132 21119645 PMC3065348

[B18] Parida A. K. Sekhar S. Panda B. B. Sahu G. Shaw B. P. (2022). Effect of panicle morphology on grain filling and rice yield: Genetic control and molecular regulation. Front. Genet. 13. doi: 10.3389/fgene.2022.876198 PMC9127237 35620460

[B19] Peng S. Khush G. S. Virk P. Tang Q. Zhou Y. (2008). Progress in ideotype breeding to increase rice yield potential. Field Crop Res. 108, 32–38. doi: 10.1016/j.fcr.2008.04.001

[B20] Seki M. Feugier F. G. Song X. J. Ashikari M. Nakamura H. Ishiyama K.. (2011). A mathematical model of phloem sucrose transport as a new tool for designing rice panicle structure for high grain yield. Plant Cell Physiol. 56, 605–619. doi: 10.1093/pcp/pcu191 25516572

[B21] Terao T. Nagata K. Morino K. Hirose T. (2010). A gene controlling the number of primary rachis branches also controls the vascular bundle formation and hence is responsible to increase the harvest index and grain yield in rice. Theor. Appl. Genet. 120, 875–893. doi: 10.1007/s00122-009-1218-8 20151298

[B22] Wood S. N. (2011). Fast stable restricted maximum likelihood and marginal likelihood estimation of semiparametric generalized linear models. J. R. Stat. Soc B. 73, 3–36. doi: 10.1111/j.1467-9868.2010.00749.x

[B23] Xing Y. Zhang Q. (2010). Genetic and molecular bases of rice yield. Annu. Rev. Plant Biol. 61, 421–442. doi: 10.1146/annurev-arplant-042809-112209 20192739

[B24] Xu Z. Chen W. Zhang L. Yang S. (2005). Design principles and parameters of rice ideal panicle type. Chin. Sci. Bull. 50, 2253–2256. doi: 10.1007/BF03182678

[B25] Yabe S. Yoshida H. Kajiya-Kanegae H. Yamasaki M. Iwata H. Ebana K.. (2018). Description of grain weight distribution leading to genomic selection for grain-filling characteristics in rice. PloS One 13, e0207627. doi: 10.1371/journal.pone.0207627 30458025 PMC6245794

[B26] Yamaki S. Miyabayashi T. Eiguchi M. Kitano H. Nonomura K. I. Kurata N. (2010). Diversity of panicle branching patterns in wild relatives of rice. Breed. Sci. 60, 586–596. doi: 10.1270/jsbbs.60.586

[B27] Yamamoto E. Takashi T. Morinaka Y. Lin S. Kitano H. Matsuoka M.. (2007). Interaction of two recessive genes, *hbd2* and *hbd3*, induces hybrid breakdown in rice. Theor. Appl. Genet. 115, 187–194. doi: 10.1007/s00122-007-0554-9 17487470

[B28] Yang J. Zhang J. (2010). Grain-filling problem in 'super' rice. J. Exp. Bot. 61, 1–5. doi: 10.1093/jxb/erp348 19959608

[B29] Yoshinaga S. Takai T. Arai-Sanoh Y. Ishimaru T. Kondo M. (2013). Varietal differences in sink production and grain-filling ability in recently developed high-yielding rice (*Oryza sativa* l.) varieties in Japan. Field Crop Res. 150, 74–82. doi: 10.1016/j.fcr.2013.06.004

[B30] Yoshioka Y. Iwata H. Ohsawa R. Ninomiya S. (2005). Quantitative evaluation of the petal shape variation in *Primula sieboldii* caused by breeding process in the last 300 years. Heredity 94, 657–663. doi: 10.1038/sj.hdy.6800678 15829983

