# Supplementary material for: Independent control of organ number and distribution pattern in rice panicle
Source: Front Plant Sci. 2023 Feb 7;14:1119770. doi: 10.3389/fpls.2023.1119770 (PMC9941638; doi:10.3389/fpls.2023.1119770)
Supplement: SUPPLEMENTARY DATA SHEET 3 — An R script used in this study. [file DataSheet_3.pdf]

```
#### arguments ####
geno.filename = "Data Sheet 1.CSV"
panicle.filename = "Data Sheet 2.CSV"
ordinary.traits = c("rachisLength", "nTotalSpikelet", "nPrimaryBranch",
 "totalPrimaryBranchLength", "nSecondaryBranch")
traits = c("nSp", "nSB", "lPB")

#### (1) read data ####
geno = read.csv(geno.filename)
data = read.csv(panicle.filename)
data = data[data$exp == "BIL", ]

#### (2) rearrange data ####
dataSet = data.frame(gid=NULL, indiv=NULL, totalPrimaryBranchLength=NULL,
 rachisLength=NULL,
                     nTotalSpikelet=NULL, nPrimaryBranch=NULL,
 nSecondaryBranch=NULL,
                     PB=NULL, nG=NULL, nSB=NULL, lenPB=NULL)
for(i in unique(data$gid)){
  datai = data[data$gid == i, ]
  for(j in unique(datai$indiv)){
    dataj = datai[datai$indiv == j, ]
    if(nrow(dataj) != 1) stop("The dataj is wrong!")
    dataSetAdd = data.frame(gid = rep(i, dataj$Primary_branch_number),
                            indiv = rep(j, dataj$Primary_branch_number),
                            totalPrimaryBranchLength =
 rep(dataj$Total_primary_branch_length, dataj$Primary_branch_number),
                            rachisLength = rep(dataj$Rachis_length,
 dataj$Primary_branch_number),
                            nPrimaryBranch = rep(dataj$Primary_branch_number,
 dataj$Primary_branch_number),
                            nSecondaryBranch =
 rep(dataj$Total_secondary_branch_number, dataj$Primary_branch_number),
                            nTotalSpikelet = rep(dataj$Total_spikelet_number,
 dataj$Primary_branch_number),
                            PB = 1:dataj$Primary_branch_number,
                            nSp = as.vector(as.matrix(dataj[,
 which(as.character(names(data)) ==
 "Spikelet_number_on_primary_branch_1"):which(as.character(names(data))
 == paste("Spikelet_number_on_primary_branch_",
 dataj$Primary_branch_number, sep=""))])),
                            nSB = as.vector(as.matrix(dataj[,
 which(as.character(names(data)) ==
```

```
 "Secondary_branch_number_on_primary_branch_1"):which(as.character(names(
 data)) == paste("Secondary_branch_number_on_primary_branch_",
 dataj$Primary_branch_number, sep=""))])),
                            lPB = as.vector(as.matrix(dataj[,
 which(as.character(names(data)) ==
 "Primary_branch_length_1"):which(as.character(names(data)) ==
 paste("Primary_branch_length_", dataj$Primary_branch_number,
 sep=""))])))
    dataSet = rbind(dataSet, dataSetAdd)
  }
}
rm(datai, dataj, dataSetAdd, i, j, data) ; gc()
stan.PB = rep(NA, nrow(dataSet))
for(i in unique(dataSet$gid)){
  datai = dataSet[dataSet$gid == i, ]
  for(j in unique(datai$indiv)){
    dataj = datai[datai$indiv == j, ]
    PBj = dataj$PB
    nPB = dataj$nPrimaryBranch[1]
    if(nPB != max(PBj)){
      print(paste(i, j, "nPB differs from max(PBj).", sep=" - "))
      nPB = max(PBj)
    }
    stan.PB[dataSet$gid == i & dataSet$indiv == j] = (PBj - 1)/(nPB - 1)
  }
}
dataSet$stan.PB = stan.PB
rm(i, j, datai, dataj, nPB, PBj, stan.PB) ; gc()
write.csv(dataSet, "dataSet.csv", row.names = F)

#### (3) choose df for spline ####
try = min(dataSet$nPrimaryBranch) - 1
dfs = numeric(length(traits))
names(dfs) = traits
for(trait in traits){
  print(paste("============", trait , "==============="))
  gcv.cumsum = rep(0, try)
  dataNow = data.frame(gid=dataSet$gid,
                       indiv=dataSet$indiv,
                       stan.PB=dataSet$stan.PB,
                       trait=dataSet[, as.character(names(dataSet)) ==
 trait])
  for(i in unique(dataNow$gid)){
```

```
    datai = dataNow[dataNow$gid == i, ]
    k = AIC = GCV = rep(NA, try)
    for(df in 3:try){
      model = mgcv::gam(trait ~ s(stan.PB, bs="cr", k=df, fx=F),
 data=datai, knots=list(stan.PB=seq(0, 1, length.out=df)))
      k[df] = df
      AIC[df] = model$aic
      GCV[df] = summary(model)$sp.criterion
    }
    select = data.frame(gid=rep(i, try), base=rep("cr", try), k=k,
 AIC=AIC, GCV=GCV)
    gcv.cumsum = gcv.cumsum + select$GCV
  }
  best = data.frame(a=select$k, c=rank(gcv.cumsum)) # , c=gcv.cumsum)
  print(best[which(best$c == min(best$c, na.rm=T) & !is.na(best$c)),])
  dfs[names(dfs)==trait] = which.min(best$c)
}
rm(best, datai, dataNow, model, select, AIC, df, GCV, gcv.cumsum, i, k,
 trait, try) ; gc()

#### (4) get spline ####
require(mgcv)
base = "cr"
dat = c()
models = vector(mode = "list", length = length(traits)) ; names(models) =
 traits
for(trait in traits){
  for(i in unique(dataSet$gid)){
    datai = dataSet[dataSet$gid == i, ]
    mat = rown = NULL
    dataNow = data.frame(gid = datai$gid,
                         indiv = datai$indiv,
                         stan.PB = datai$stan.PB,
                         trait = datai[, as.character(names(datai)) ==
 trait])
    model = mgcv::gam(trait ~ s(stan.PB, bs = base, k = dfs[traits ==
 trait], fx = F), data = dataNow,
                      knots=list(stan.PB = seq(0, 1, length.out=dfs[traits ==
 trait])))
    models[[trait]][[i]] = model
    mati = predict(model, type = "lpmatrix")
    if(is.null(mat)){
      mat = mati
```

```
      rown = which(dataSet$gid == i)
    }else{
      mat = rbind(mat, mati)
      rown = c(rown, which(dataSet$gid == i))
    }
  }
  for(i in 2:(dfs[traits == trait])){
    datai = data.frame(trait = trait, b = i, x = dataSet$stan.PB[rown], y
 = mat[, i])
    datai = datai[order(datai$x), ]
    dat = rbind(dat, datai)
  }
}
rm(datai, dataNow, mat, mati, model, i, rown) ; gc()
dat$b = factor(dat$b)
dat$trait[is.element(dat$trait, "lPB")] = "Primary branch length"
dat$trait[is.element(dat$trait, "nSp")] = "Spikelet"
dat$trait[is.element(dat$trait, "nSB")] = "Secondary branch"
gp = ggplot2::ggplot(data = dat, ggplot2::aes(x = x, y = y, color = b)) +
  ggplot2::facet_wrap(. ~ trait, nrow = length(traits)) +
  ggplot2::geom_smooth(method = "loess", span = 0.2, size = 0.6, se =
 FALSE) +
  ggplot2::labs(x="PB (0=distal; 1=proximal)",
 y=expression(italic(b[i])(x))) +
  ggplot2::theme_void() +
  ggplot2::theme(plot.margin = ggplot2::unit(c(2, 5.5, 2, 2), "mm")) +
  ggplot2::theme(strip.text = ggplot2::element_text(face = "bold", size
 =10)) +
  ggplot2::theme(axis.title = ggplot2::element_text(size = 10)) +
  ggplot2::theme(axis.text = ggplot2::element_text(colour = "black", size
 = 9)) +
  ggplot2::theme(legend.position = 'none') +
  ggplot2::theme(panel.background = ggplot2::element_rect(fill =
 "transparent", color = NA),
              plot.background = ggplot2::element_rect(fill =
 "transparent", color = NA),
              legend.background = ggplot2::element_rect(fill =
 "transparent", colour = NA),
              legend.key = ggplot2::element_rect(fill = "transparent",
 colour = NA),
              legend.box.background = ggplot2::element_rect(fill =
 "transparent", colour = NA))
ggplot2::ggsave(filename="basis_functions.png", plot = gp, dpi = 1200,
```

```
 width = 2.4, height = 3, bg = "transparent")
rm(gp, dat, base) ; gc()

#### (5) make data for ordinary traits ####
dataAna = data.frame(gid=unique(dataSet$gid))
for(trait in ordinary.traits){
  obs = rep(NA, nrow(dataAna))
  for(i in 1:nrow(dataAna)){
    obs[i] = mean(dataSet[dataSet$gid == dataAna$gid[i],
 as.character(names(dataSet)) == trait], na.rm = TRUE)
  }
  dataAna = data.frame(dataAna, obs = obs)
  names(dataAna)[ncol(dataAna)] = trait
}
rm(i, obs) ; gc()

#### (6) make data for distribution pattern ####
require(mgcv)
for(trait in traits){
  obs = matrix(NA, nrow = nrow(dataAna), ncol = dfs[traits == trait])
  for(i in 1:nrow(dataAna)){
    model = models[[trait]][[dataAna$gid[i]]]
    x = seq(0, 1, by = 0.01)
    x = mean(diff(x)) / 2 + x
    x = x[-length(x)]
    resForInt = predict(model, data.frame(stan.PB = x), type = "response")
    int = 0
    for(j in 1:length(resForInt)) int = int + mean(diff(x)) * resForInt[j]
    obs[i, ] = model$coefficients / int
  }
  dataAna = data.frame(dataAna, obs)
  names(dataAna)[(ncol(dataAna) - dfs[traits == trait] +
 1):ncol(dataAna)] = paste0(trait, "_", 1:dfs[traits == trait])
}
rm(model, obs, i, j, int, x, resForInt) ; gc()
dpfun = vector(mode = "list", length = length(traits))  # function for
 PCA
names(dpfun) = traits
scoreData = data.frame(gid = dataAna$gid)
plotPCA = c()
n.plotPCA = 2
for(trait in traits){
  print(paste("=============", trait , "==============="))
```

```
 mat = rown = NULL
 for(i in unique(dataSet$gid)){
   model = models[[trait]][[i]]
   mati = predict(model, type = "lpmatrix")
   if(is.null(mat)){
     mat = mati
     rown = which(dataSet$gid == i)
   }else{
     mat = rbind(mat, mati)
     rown = c(rown, which(dataSet$gid == i))
   }
 }
 # data
 dataNow <- dataAna[, substr(as.character(names(dataAna)), 1,
nchar(trait)) == trait]
 rownames(dataNow) = dataAna$gid
 # PCA
 pca = prcomp(as.matrix(dataNow))
 rot = pca$rotation
 if (trait == "lPB") rot = rot * -1
 rot.inv = t(rot)
 if (trait == "lPB") pca$x = pca$x * -1
 dpfun[[trait]]$x = pca$x
 dpfun[[trait]]$center = pca$center
 dpfun[[trait]]$rot.inv = rot.inv
 dpfun[[trait]]$mat = mat
 dpfun[[trait]]$rown = rown
 # PVE
 pve = unlist(summary(pca)[1])^2
 pve = pve / sum(pve)
 print(pve)
 write.csv(pve, paste0("", trait, "_PC.pve.csv"))
 rm(pve) ; gc()
 # PCA effect
 for(i in 1:n.plotPCA){
   score = matrix(0, 3, dfs[traits == trait])
   print(c(-sd(pca$x[, i])*2, 0, sd(pca$x[, i])*2))
   score[, i] = c(-sd(pca$x[, i])*2, 0, sd(pca$x[, i])*2)
   coef.pc = score %*% rot.inv + matrix(rep(pca$center, 3), nrow = 3,
byrow = T)
   rownames(coef.pc) = c("-2sd", "mean", "2sd")
   z = (as.matrix(mat)[, 1:(dfs[traits == trait])]) %*% t(coef.pc)    #
calculate PC's feature
```

```
    for(j in 1:3) {
      datai = data.frame(trait = trait, pc = paste0("PC", i), lab =
 rownames(coef.pc)[j], x = dataSet$stan.PB[rown], y = z[, j])
      plotPCA = rbind(plotPCA, datai)
    }
  }
  # save score
  scoreDataAdd = data.frame(pca$x)
  names(scoreDataAdd) = paste0(trait, "_PC", 1:ncol(pca$x))
  scoreData = cbind(scoreData, scoreDataAdd)
}
rm(score, coef.pc, z, i, j, datai) ; gc()
save(dpfun, file="dpfun.RData") # function for PCA
dataAna = cbind(dataAna, scoreData[, -1])
rm(dataNow, mat, mati, model, models, pca, rot, rot.inv, scoreDataAdd,
 scoreData, rown, dfs, dataSet, trait); gc()
plotPCA$lab = factor(plotPCA$lab, levels = c("mean", "-2sd", "2sd"))
plotPCA$trait[is.element(plotPCA$trait, "lPB")] = "Primary branch"
plotPCA$trait[is.element(plotPCA$trait, "nSp")] = "Spikelet"
plotPCA$trait[is.element(plotPCA$trait, "nSB")] = "Secondary branch"
plotPCA$title = paste(plotPCA$trait, plotPCA$pc, sep = " : ")
for (trait in unique(plotPCA$trait)) {
  dat = plotPCA[is.element(plotPCA$trait, trait), ]
  gp = ggplot2::ggplot(data=dat, ggplot2::aes(x = x, y = y, col = lab,
 linetype = lab)) +
    ggplot2::facet_wrap(. ~ title, nrow = n.plotPCA, scales = "free_y") +
    ggplot2::geom_smooth(method = "loess", span = 1, size = 0.6, se =
 FALSE) +
    ggplot2::labs(x="PB (0=distal; 1=proximal)", y="") +
    ggplot2::theme_bw() +
    ggplot2::theme(plot.margin = ggplot2::unit(c(2, 5.5, 2, 2), "mm")) +
    ggplot2::theme(strip.text = ggplot2::element_text(face = "bold", size
 =9)) +
    ggplot2::theme(axis.title = ggplot2::element_text(size = 9)) +
    ggplot2::theme(axis.text = ggplot2::element_text(colour = "black",
 size = 9)) +
    ggplot2::scale_color_manual(values = c("grey35", "#0055FF",
 "#E7B800")) +
    ggplot2::scale_linetype_manual(values = c("solid", "longdash",
 "twodash")) +
    ggplot2::theme(legend.position = 'none') +
    ggplot2::theme(panel.background = ggplot2::element_rect(fill =
 "transparent", color = NA),
```

```
                  plot.background = ggplot2::element_rect(fill =
"transparent", color = NA),
                  legend.background = ggplot2::element_rect(fill =
"transparent", colour = NA),
                  legend.key = ggplot2::element_rect(fill = "transparent",
colour = NA),
                  legend.box.background = ggplot2::element_rect(fill =
"transparent", colour = NA))
  ggplot2::ggsave(filename = paste0("", trait, "_PC.png"), plot = gp, dpi
= 1200, width = 2.4, height = 2.13, bg = "transparent")
}
rm(plotPCA, gp, dat) ; gc()

shape.values = c(4, 15, 19)
color.values = c("grey35", "#0055FF", "#E7B800")
for (trait in traits) {
  pve = read.csv(paste0("", trait, "_PC.pve.csv"))$x * 100
  x = dpfun[[trait]]$x
  Category = rep("BIL", nrow(x))
  Category[is.element(rownames(x), "9000")] = "Koshi"
  Category[is.element(rownames(x), "9999")] = "Haba"
  dat = data.frame(Category = Category, PC1 = x[, 1], PC2 = x[, 2], trait
= trait)
  dat$trait[is.element(dat$trait, "lPB")] = "Primary branch"
  dat$trait[is.element(dat$trait, "nSp")] = "Spikelet"
  dat$trait[is.element(dat$trait, "nSB")] = "Secondary branch"
  gp = ggplot2::ggplot(dat, ggplot2::aes(x = PC1, y = PC2, col =
Category, fill= Category, shape = Category)) +
    ggplot2::geom_point(size = 2) +
    ggplot2::facet_wrap(. ~ trait, nrow = 1) +
    ggplot2::labs(x = paste("PC1 (", round(pve[1], digits = 1), "%)", sep
= ""),
                 y = paste("PC2 (", round(pve[2], digits = 1), "%)", sep =
"")) +
    ggplot2::theme_bw() +
    ggplot2::theme(strip.text = ggplot2::element_text(face = "bold", size
=10)) +
    ggplot2::theme(axis.title = ggplot2::element_text(size = 10)) +
    ggplot2::theme(axis.text = ggplot2::element_text(colour = "black",
size = 9)) +
    ggplot2::scale_shape_manual(values = shape.values) +
    ggplot2::scale_color_manual(values = color.values) +
    ggplot2::scale_fill_manual(values = color.values) +
```

```
   ggplot2::theme(legend.position = 'none') +
   ggplot2::theme(panel.background = ggplot2::element_rect(fill =
 "transparent", color = NA),
                  plot.background = ggplot2::element_rect(fill =
 "transparent", color = NA),
                  legend.background = ggplot2::element_rect(fill =
 "transparent", colour = NA),
                  legend.key = ggplot2::element_rect(fill = "transparent",
 colour = NA),
                  legend.box.background = ggplot2::element_rect(fill =
 "transparent", colour = NA))

  ggplot2::ggsave(filename = paste0("BIL_", trait, "_PC.png"), plot = gp,
 dpi = 1200, width = 2.4, height = 2.1)
}
rm(shape.values, color.values, pve, x, gp, n.plotPCA, dat, trait,
 Category, dpfun) ; gc()

#### (7) make data for QTL analysis ####
pheno = as.matrix(dataAna)[as.numeric(rownames(dataAna)) < 100, -1]
pheno = rbind(matrix("", nrow = 2, ncol = ncol(pheno)), pheno)
write.csv(cbind(pheno, geno), "cross.csv", row.names = F)
rm(dataAna, geno, pheno) ; gc()

#### (8) QTL analysis ####
require(qtl)
mydata = read.cross(format = "csv", file = "cross.csv", crosstype =
 "riself")
mydata = calc.genoprob(mydata, step = 2.5)
traits.ana = colnames(mydata$pheno)#[-c(6:19, 22:24, 27:29, 32:33)]
for (trait in traits.ana) {
  print(trait)
  s = scanone(mydata, pheno.col = which(colnames(mydata$pheno) == trait))
  set.seed(999)
  perm = scanone(mydata, n.perm = 1000)
  png(filename = paste0(trait, "_scanone.png"), width = 4800, height =
 1600, res = 532, bg = "transparent")
  par(mar=c(3.2, 3.2, 1.5, 0.5), mgp=c(2.0, 0.7, 0))
  plot(s, main=trait, xlab="Chromosome", ylab="LOD score", axes=F,
 col="darkblue")
  abline(h=0)
  axis(2)
  abline(h=summary(perm)[1:2], lty = c(1, 2), col = "red")
```

```
  dev.off()
  res = list(s = s, perm = perm)
  save(res, file = paste0(trait, "_scanone.RData"))
}
rm(mydata, perm, s, traits.ana, res, trait) ; gc()
```
